# The braincase of *Malawisaurus dixeyi* (Sauropoda: Titanosauria): A 3D reconstruction of the brain endocast and inner ear

**DOI:** 10.1371/journal.pone.0211423

**Published:** 2019-02-13

**Authors:** Kate A. Andrzejewski, Michael J. Polcyn, Dale A. Winkler, Elizabeth Gomani Chindebvu, Louis L. Jacobs

**Affiliations:** 1 Roy M. Huffington Department of Earth Sciences, Southern Methodist University, Dallas, Texas, United States of America; 2 Ministry of Civic Education, Culture and Community Development, Lilongwe, Malawi; State Museum of Natural History, GERMANY

## Abstract

A braincase of the Cretaceous titanosaurian sauropod *Malawisaurus dixeyi*, complete except for the olfactory region, was CT scanned and a 3D rendering of the endocast and inner ear was generated. Cranial nerves appear in the same configuration as in other sauropods, including derived features that appear to characterize titanosaurians, specifically, an abducens nerve canal that passes lateral to the pituitary fossa rather than entering it. Furthermore, the hypoglossal nerve exits the skull via a single foramen, consistent with most titanosaurians, while other saurischians, including the basal titanosauriform, *Giraffatitan*, contain multiple rootlets. The size of the vestibular labyrinth is smaller than in *Giraffatitan*, but larger than in most derived titanosaurians. Similar to the condition found in *Giraffatitan*, the anterior semicircular canal is larger than the posterior semicircular canal. This contrasts with more derived titanosaurians that contain similarly sized anterior and posterior semicircular canals, congruent with the interpretation of *Malawisaurus* as a basal titanosaurian. Measurements of the humerus of *Malawisaurus* provide a body mass estimate of 4.7 metric tons. Comparison of body mass to radius of the semicircular canals of the vestibular labyrinth reveals that *Malawisaurus* fits the allometric relationship found in previous studies of extant mammals and *Giraffatitan brancai*. As in *Giraffatitan*, the anterior semicircular canal is significantly larger than is predicted by the allometric relationship suggesting greater sensitivity and slower movement of the head in the sagittal plane.

## Introduction

Cretaceous titanosaurid material recovered from Malawi, Africa, was first described by Haughton (1928) and named *Gigantosaurus dixeyi* based on presumed similarity to specimens collected in Tanzania that were referred to as *Gigantosaurus* [1, 2]. However, the generic name ‘*Gigantosaurus’* was preoccupied and was replaced with the generic name *Tornieria* by Sternfeld (1911) and *G*. *dixeyi* from Malawi became known as *Tornieria dixeyi* without further justification [3]. The generic name *Tornieria* was later changed to *Janenschia* by Wild (1991) [4]. Because the taxon from Malawi is distinct from the titanosaurid genus *Janenschia*, which was recovered from Jurassic beds in Tanzania, a new generic name, *Malawisaurus*, was erected by Jacobs et al. (1993) to accommodate the titanosaurid species from Malawi [5].

First described by Haughton (1928), *Malawisaurus dixeyi* was later redescribed by Jacobs et al. (1993) and Gomani (2005) after field expeditions by the Malawi Dinosaur Project (MDP) in 1984, 1987, 1989, 1990, and 1992 recovered new fossil material in the same Dinosaur Beds near Mwakasyunguti, Karonga District, northern Malawi [5, 6]. The Dinosaur Beds are estimated to be Early Cretaceous (Aptian) age based on biochronology [7] and regional proximity to carbonatites to the north and south of the study area dated to 123± 3 to 111± 13.1Ma using K-Ar dating methods [8, 9]; however, Le Loeuff et al. (2012) argue the Dinosaur Beds of Malawi could be Late Cretaceous in age based on the vertebrate assemblage and suggest more evidence is needed to accurately date these sites [10]. Field expeditions by the Malawi Dinosaur Project recovered a nearly complete basicranium, Mal-202-1, of *Malawisaurus dixeyi* and associated parietals, ectopterygoid, quadrate, cervical vertebrae, and post cranial elements [6]. The goals of this study are to offer a detailed description of the braincase, digital reconstructions of the endocast and inner ear based on CT scanning and compare these data to other sauropods to test the phylogenetic position of *Malawisaurus* among titanosaurs. Additionally, the study provides an estimation of the body mass of *Malawisaurus dixeyi*.

## Materials and methods

The material described, Mal 202–1, is currently on loan to the Shuler Museum of Paleontology at Southern Methodist University (SMU) Dallas, Texas and will be returned to the paleontological collection of the Malawi Department of Antiquities Lilongwe, Malawi. To produce a three-dimensional reconstruction of the endocast and inner ear, the specimen, Mal 202–1, was scanned at the University of Texas High Resolution X-ray CT facility using a voltage of 200kV and a current of 0.12mA producing 1707 slices with a voxel size of 70.6μm. Data from the scan were imported into Amira v 4.2 for analysis and visualization. The model was then imported into MeshLab where a laplacian smoothing algorithm was applied. Final rendering was completed in LightWave.

Measurements of the semicircular canals were conducted in Amira v 4.2 following protocols from Spoor and Zonneveld (1995) [11]. Radii of the semicircular canals of the vestibular labyrinth was quantified by the radius of curvature, or half the average of the arc height and width [12]. Body mass of *Malawisaurus* was calculated using the following regression from Campione and Evans (2012), based on the circumference of the humerus, Mal-221, found associated with the basicranium:

$$\text{log}\;(BM)\;=\;2.6861*\;Log(C_{H})-0.1438$$

where BM is body mass in grams and C_H_ is circumference of the humerus in mm [13].

## Osteology

**Fig 1:**
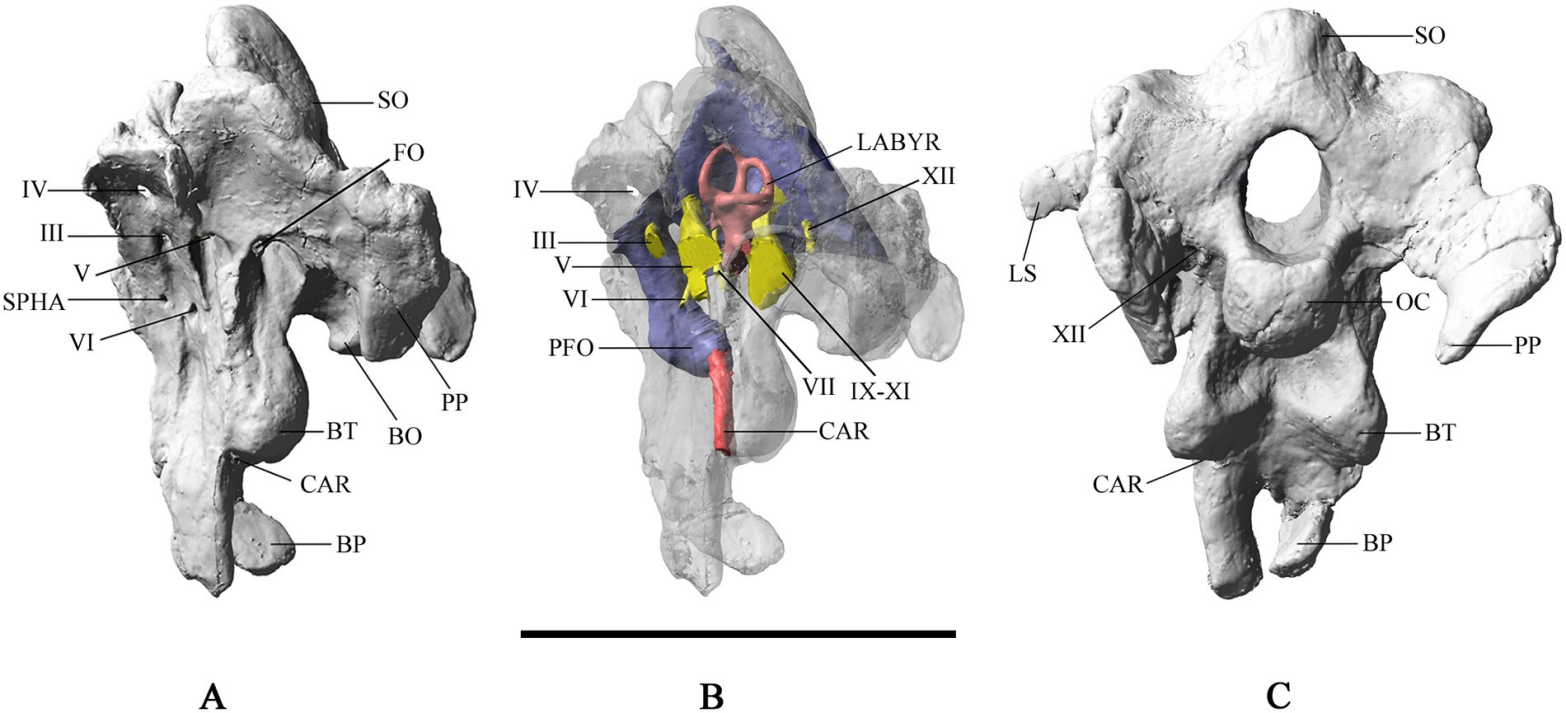
Braincase of *Malawisaurus dixeyi*. (A) lateral view; (B) lateral view with endocast; (C) posterior view. Abbreviations: BO, basioccipital; BP, basipterygoid process; BT, basal tuber; CAR, canal for cerebral carotid artery; FO, fenestra ovalis; LABYR, labyrinth; LS, laterosphenoid; OC, occipital condyle; PFO, pituitary fossa; PP, paroccipital process; SO, supraoccipital; SPHA, canal for sphenopalatine artery; III, oculomotor nerve; IV, trochlear nerve; V, trigeminal nerve; VI, abducens nerve; VII, facial nerve; IX-XI, shared canal for glossopharyngeal, vagus, and spinal accessory nerves; XII, hypoglossal nerve. Scale bar equals 10cm.

The bones surrounding the brain of *Malawisaurus* (Mal-202-1) are well preserved, but with anterior portions missing from the frontals and parietals (Fig 1). Bones present are completely ossified, and sutures are indistinct both optically and in CT data, suggesting the specimen represents a mature or adult individual. The braincase is exceptionally well preserved, but with a small amount of shear, the left side shifted slightly anterior relative to the right, exposing the internal structure of the endocranial cavity.

The supraoccipital forms the dorsal margin of the foramen magnum and bears a prominent nuchal crest that reaches maximum prominence at the dorsal edge, which presumably would contact the parietal as observed in *Giraffatitan* [14]. The wings of the supraoccipital flank the nuchal crest and are concave forming bilateral depressions. The exoccipital and opisthotic are co-ossified into a single complex (otoccipital), which forms the lateral margin of the foramen magnum. The otoccipitals form large anteroposteriorly flattened wing-like paroccipital processes that curve ventrally. A ventrally directed depression for the quadrate articulation lies on the posterolateral surface of the paroccipital processes. The otoccipital contains the mediolaterally narrow and elongate metotic foramen (= vagal, jugular foramen). The metotic foramen served as a passageway for the glossopharyngeal, vagus, and accessory nerves (cranial nerves IX-XI).

The foramen magnum is ovoid, taller (28mm) than wide (20mm), and slightly narrower than the occipital condyle (30mm). With the supraoccipital oriented vertically, the occipital condyle faces posteroventrally, consistent with evidence from the inner ear suggesting a habitual head posture near horizontal or with a slightly downturned muzzle, similar to the condition seen in *Camarasaurus* [15]. However, Taylor et al., (2009), citing head posture in extant animals, argued that the planar orientation of the lateral semicircular canal may not accurately reflect habitual posture [16]. The basal tubera of the basioccipital are linked to the condylar region by two thick ridges that outline the subcondylar recess, as in *Sarmientosaurus* [17], *Muyelensaurus* [18], and the Uzbekistan titanosaur CCMGE 628/12457 [19].

Beneath the ridge of the basal tubera, the canals for the cerebral carotid arteries enter the braincase near the base of the basipterygoid processes. The bases of the basipterygoid processes are closely spaced. The basipterygoid processes are parallel as in *Sarmientosaurus* and *Muyelensaurus*, unlike the condition observed in *Camarasaurus* [20], *Giraffatitan*, and *Jainosaurus* [21], in which the processes diverge widely.

## Cranial endocast

**Fig 2:**
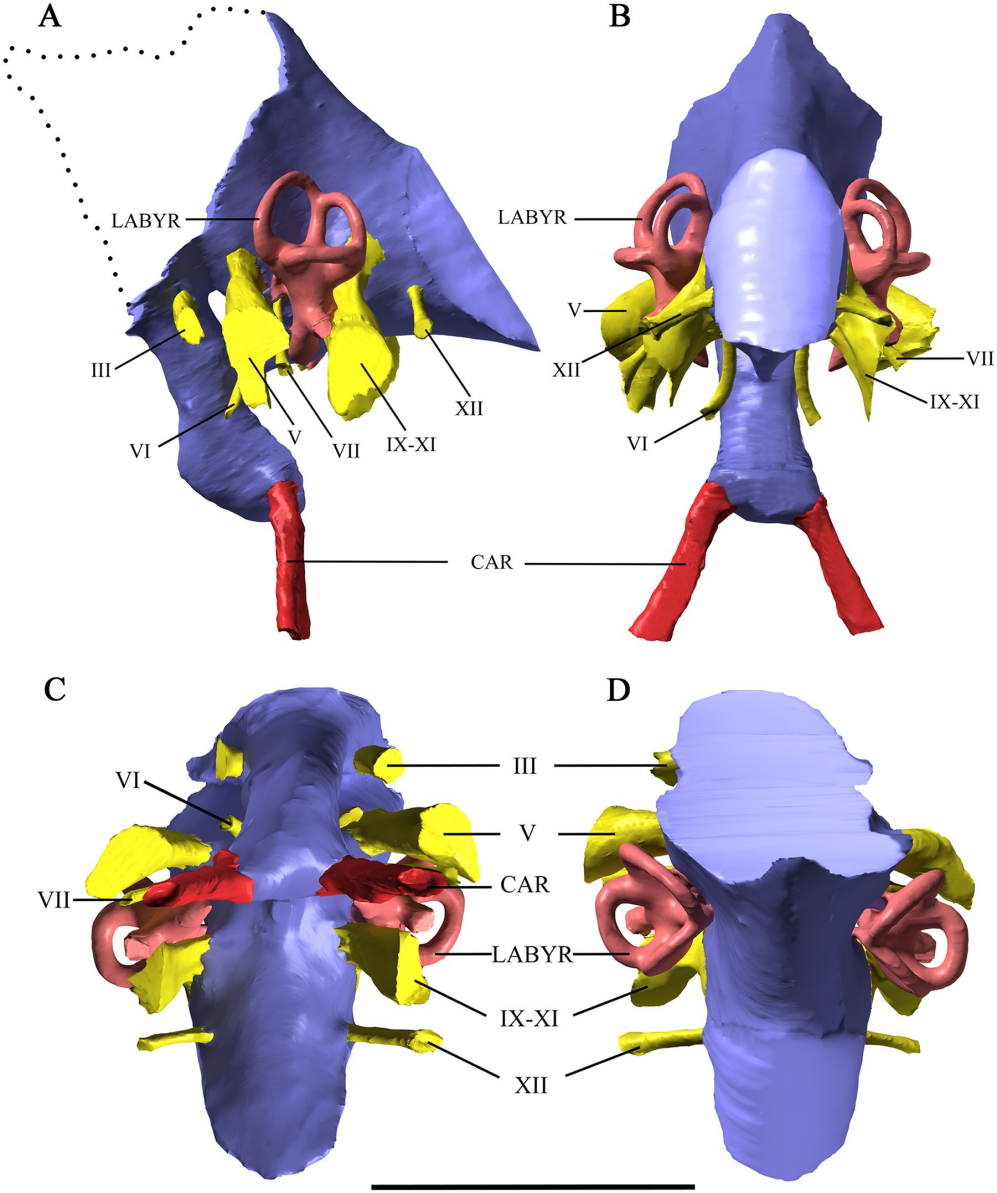
Cranial endocast and vestibular labyrinth of *Malawisaurus dixeyi*. (A) left lateral view; (B) caudal view; (C) ventral view; (D) dorsal view; dashed line represents reconstruction of full endocast based on the endocast of *Sarmientosaurus*. Endocast represented by purple coloring; cranial nerves by yellow coloring; vestibular labyrinth by pink coloring; carotid artery by red coloring. Scale bar equals 5cm.

The braincase of *M*. *dixeyi* is well preserved with little distortion allowing for clear resolution of the internal anatomy. The digitally reconstructed endocast lacks the olfactory and cerebral regions (Fig 2). Characteristic traits of sauropods observed in the endocast include the presence of a well-defined and large pituitary fossa and the lack of distinction of gross regions of the brain, presumably obscured by the presence of overlying thick meninges and extensive venous sinuses in life [20, 22, 23, 24, 25].

The caudal dural expansion, a prominent venous feature of sauropods [20, 23], is not preserved in this specimen, although based on the sharp posterior-anterior rise on the dorsal surface of the endocast, it likely had this feature. A median canal connects the pituitary space with the braincase cavity between the trigeminal and abducens nerves. This is observed in basal taxa including *Spinophorosaurus* [23] and more derived sauropod taxa including *Camarasaurus* [20] and *Jainosaurus* [17]. This canal has been proposed as a passage for the basilar artery [25] or of venous origin [19]. The canals for the cerebral carotid arteries enter the posteroventral margin of the pituitary fossa. The pituitary fossa is similar to most sauropods in lacking a ventral median canal representing the craniopharyngeal canal present in the unnamed Uzbekistan titanosaur [19].

## Cranial nerves

The cranial nerves have an arrangement similar to other sauropods. The trigeminal nerve (V) is the largest of the cranial nerves and exits caudal to the infundibular region via a single foramen. The endocast shows little evidence for the division of the trigeminal nerve into the ophthalmic (V_1_), maxillary (V_2_), and mandibular (V_3_) branches as observed in *Sarmientosaurus* [17]; however, Gomani (2005) noted anterior and posterior grooves that exit ventral to the canal and are visible on the CT scans presented here. These grooves may have held maxillary and mandibular branches. The abducens nerve (VI) originates ventral to the trigeminal nerve and extends lateral to the pituitary fossa rather than entering it, which is a derived character state for titanosaurs [17, 24, 25]. The facial nerve (VII) originates posterior to the abducens and trigeminal nerves and passes ventrolaterally. A large opening posterior to the vestibular labyrinth serves as the passageway for cranial nerves IX-XI. The hypoglossal nerve (XII) exits via one foramen, consistent with most titanosaurs; however, *Sarmientosaurus* [17], *Jainosaurus* [21], and *Pitekunsaurus* [26] contain multiple rootlets.

## Inner ear

**Fig 3:**
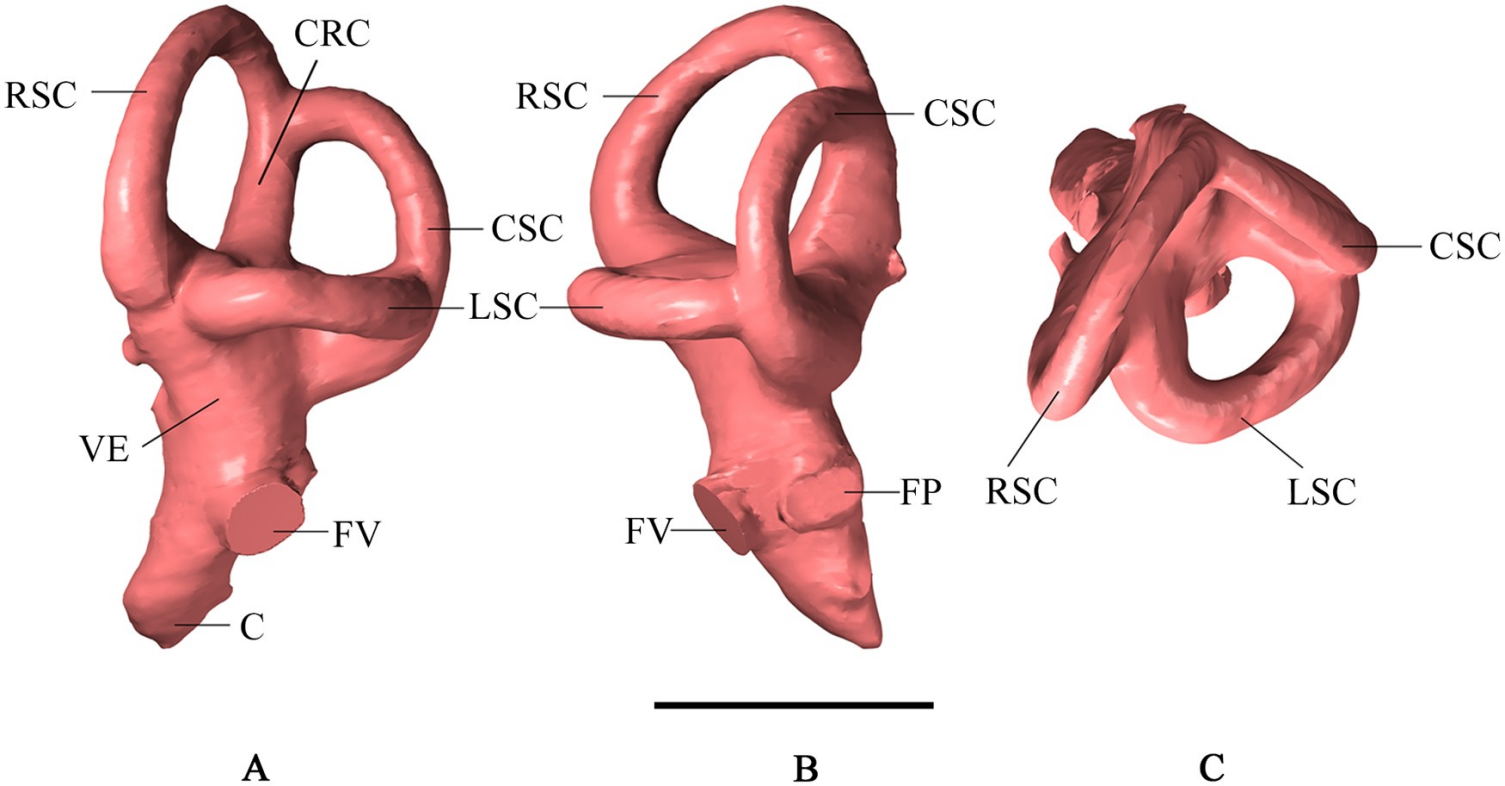
Left vestibular labyrinth of *Malawisaurus dixeyi*. (A) lateral view; (B) posterior view; (C) dorsal view. Abbreviations: C, cochlea; CRC, crus commune; CSC caudal (posterior) semicircular canal; FP, fenestra perilymphatica; FV fenestra vestibuli; LSC, lateral semicircular canal; RSC, rostral (anterior) semicircular canal; VE, vestibule of inner ear. Scale bar equals 2cm.

**Fig 4:**
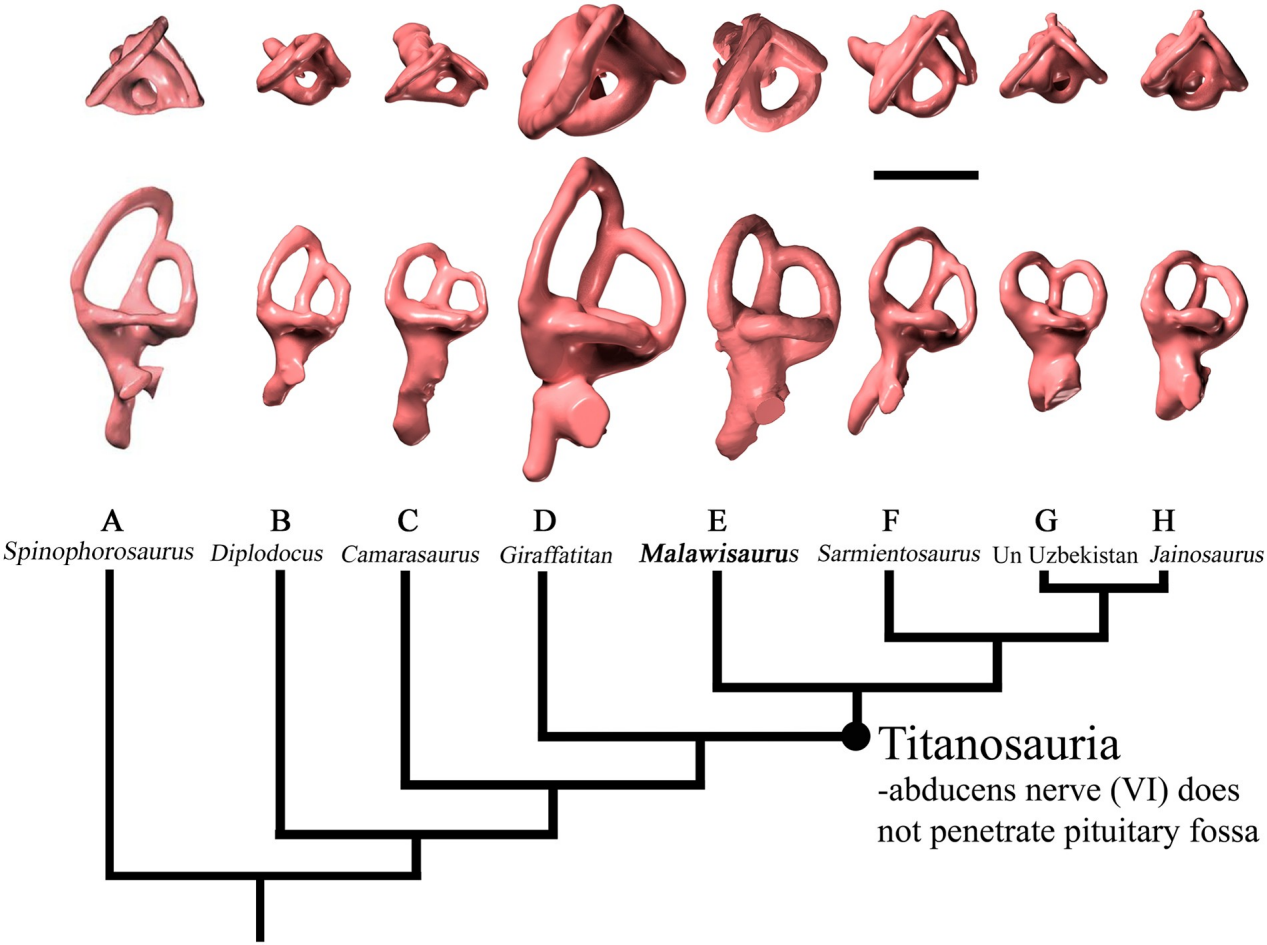
Comparison of left vestibular labyrinth of sauropod dinosaurs (modified from Knoll et al., 2012 and Martinez et al., 2016). (A) *Spinophorosaurus nigerensis* (B) *Diplodocus longus*; (C) *Camarasaurus lentus*; (D) *Giraffatitan brancai*; (E) *Malawisaurus dixeyi*; (F) *Sarmientosaurus musacchioi*; (G) Unnamed derived titanosaur from Uzbekistan (CCMGE 628/12457); (H) *Jainosaurus septentrionalis*. Scale bar equals 2cm.

The vestibular labyrinth of *M*. *dixeyi* is intermediate in size compared to the large labyrinth of *Giraffatitan* and the smaller sizes in advanced titanosaurs such as *Jainosaurus* (Figs 3 and 4). The rostral (anterior) semicircular canal is larger and is elevated dorsally compared to the caudal (posterior) semicircular canal similar to the condition observed in *Giraffatitan* (Fig 4). This supports *M*. *dixeyi* as a basal titanosaur as more advanced titanosaurs have approximately equal caudal and rostral semicircular canals. The lateral semicircular canal has the smallest diameter of the three, consistent with most sauropods; however, the lateral semicircular canal of *M*. *dixeyi* is longer and more slender in comparison to the lateral semicircular canal of other sauropods. The angle between the rostral and caudal semicircular canals is nearly orthogonal and similar to most titanosaurs except *Sarmientosaurus*.

## Body mass and semicircular dimensions

**Fig 5:**
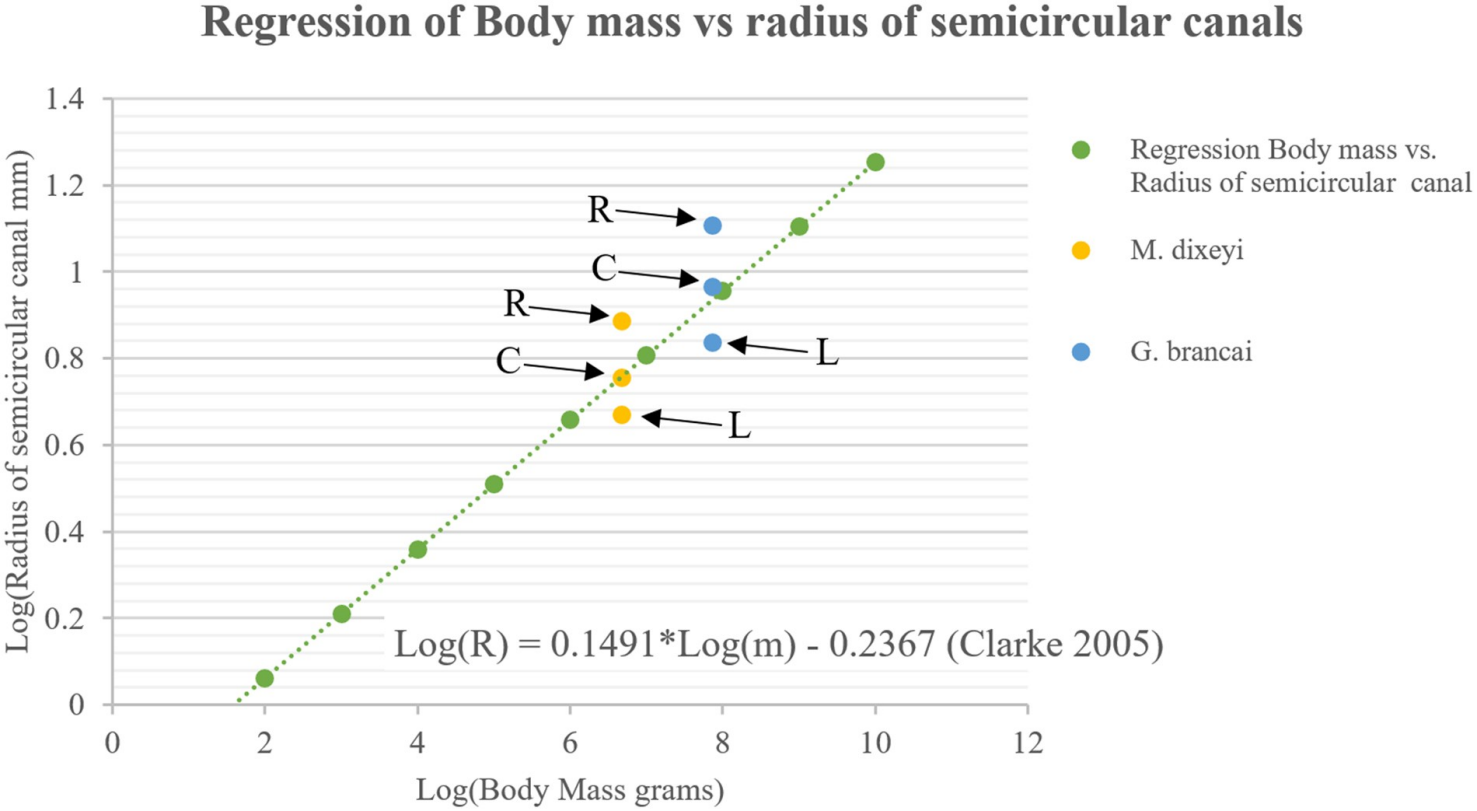
Bivariate logarithmic distribution and corresponding line of regression for the external radius of curvature (R) of semicircular canals and body mass (m). R, rostral semicircular canal; C, caudal semicircular canal; L, lateral semicircular canal. (Modified from Clarke 2005).

Using a regression from Campione and Evans (2012), the body mass of *M*. *dixeyi* was estimated to be 4.73 metric tons based on the circumference of the humerus (Mal-221) found associated with the basicranium (Table 1). Measurements for the radii of the semicircular canals are shown in Table 2. Comparison of the measured radii of the semicircular canals and the predicted radii based on a regression of body mass from Clarke (2005) reveals a pattern similar to *Giraffatitan brancai* [27] (Fig 5). The caudal semicircular canal falls within the 95% confidence interval of predicted size, but the lateral semicircular canal is smaller than the predicted size. The rostral semicircular canal is significantly larger than the predicted size.

**Table 1:** Measurements of associated humerus and body mass estimate of *Malawisaurus dixeyi* based upon the regression from Campione and Evans (2012).

Specimen Number	Humerus Length (mm)	Humerus Circumference (mm)	Predicted mass (grams)
Mal-316	730	345.5	4731285.6

**Table 2:** Measurements of semicircular canals from the braincase of *Malawisaurus dixeyi*.

Semicircular Canal	Diameter (mm)	Semicircular Canal	Diameter (mm)	Semicircular Canal	Diameter (mm)
Rostral Left	15.18	Caudal Left	11.46	Lateral Left	8.35
	14.19		11.05		9.35
	16.75		11.03		10.20
	14.13		12.36		9.40
	16.25		11.11		8.99
Rostral Right	16.02	Caudal Right	11.09	Lateral Right	8.36
	15.58		11.16		10.10
	14.85		12.32		10.07
	15.68		11.01		9.35
	14.99		11.20		9.32
Average Rostral diameter (mm)	15.36	Average Caudal diameter (mm)	11.38	Average Lateral diameter (mm)	9.35
Average Rostral radius (mm)	7.68	Average Caudal radius (mm)	5.69	Average Lateral radius (mm)	4.67

## Discussion

The lateral semicircular canal of *M*. *dixeyi* is longer and more slender compared to most sauropod taxa although *M*. *dixeyi* is a basal titanosaurian as shown by its endocranial structure consistent with other features of the skeleton [6], (Fig 4). This condition of the lateral semicircular canal is similar to *Sarmientosaurus* and may indicate increased sensitivity in the mediolateral plane emphasizing lateral scanning movements of the head and eyes [17]. The angle between the semicircular canals of *M*. *dixeyi* is nearly orthogonal. A study by Berlin et al. 2013 concluded that deviations from orthogonality of the semicircular canals in mammals was negatively correlated with vestibular sensitivity [28]. Research conducted by Malinzak et al. (2011; 2012) found mammals with the greatest deviations from canal orthogonality experienced slower head rotations during locomotion [29, 30]. Together this suggests *Malawisaurus* may have experienced higher angular head velocities during locomotion and increased vestibular sensitivity compared to *Sarmientosaurus*. Furthermore, the rostral semicircular canal *of M*. *dixeyi* is larger than the predicted size based on regression from Clarke (2005), which may indicate greater sensitivity. This condition supports behaviors including slower movement of the head in the sagittal plane [27].

## Conclusion

CT scans of the braincase of *Malawisaurus dixeyi* recovered from the Dinosaur Beds of Malawi reveal insights into the paleoneuroanatomy and physiology of a basal titanosaur. The derived character state of an abducens nerve canal that passes lateral to rather than entering the pituitary fossa places *Malawisaurus dixeyi* within Titanosauria. The disproportionate size of the semicircular canals of the vestibular labyrinth with a larger rostral semicircular canal than caudal semicircular canal supports *M*. *dixeyi* as a basal titanosaur since derived titanosaurs exhibit equally sized semicircular canals. Body mass estimates based on circumference of the humerus are similar to estimates calculated using the radius of the semicircular canals with the caudal (posterior) semicircular canal falling within the predicted mass of 4.73 metric tons. This study has revealed the potential for imaging software in identifying important characters and new insight into physiology and behavior of extinct taxa.

## Supporting information

S1 Appendix 3D model of braincase and reconstructed endocast of *Malawisaurus dixeyi*. (PDF)
