# Supplementary figures and images for: The braincase of Malawisaurus dixeyi (Sauropoda: Titanosauria): A 3D reconstruction of the brain endocast and inner ear
Source: PLoS One. 2019 Feb 13;14(2):e0211423. doi: 10.1371/journal.pone.0211423 (PMC6373922; doi:10.1371/journal.pone.0211423)

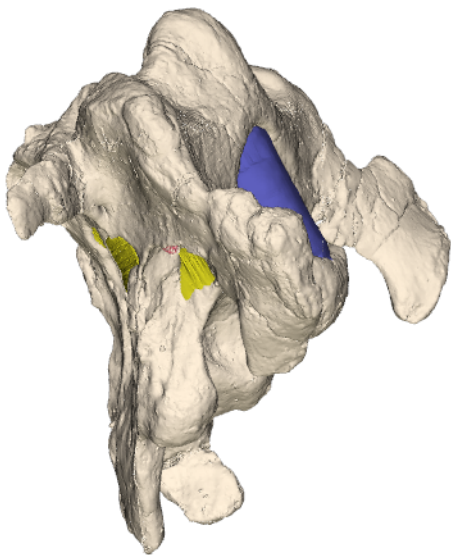

Supplement: S1 Appendix — (PDF) [file pone.0211423.s001.pdf]
